# Effect of enriched bone-marrow aspirates on the dimensional stability of cortico-cancellous iliac bone grafts in alveolar ridge augmentation

**DOI:** 10.1186/s40729-022-00435-1

**Published:** 2022-09-05

**Authors:** Hendrik Naujokat, Klaas Loger, Aydin Gülses, Christian Flörke, Yahya Acil, Jörg Wiltfang

**Affiliations:** Department of Oral and Maxillofacial Surgery, University Hospital Schleswig-Holstein (Head: Prof. Dr. Dr. Jörg Wiltfang), Arnold-Heller-Straße 3, Haus B, Kiel, Germany

**Keywords:** Bone graft resorption, Alveolar augmentation, Onlay bone graft, Iliac crest, Bone-marrow aspirate concentrate, Dental implants

## Abstract

**Background:**

The objective of the current study was to assess the clinical and radiological outcomes following autologous grafting from the iliac crest treated with autologous stem cells in-situ to reduce the postoperative bone graft resorption rate.

**Materials and methods:**

The study group consisted of patients who underwent vertical augmentation of the jaws via bone grafts harvested from the iliac crest enriched with bone-marrow aspirate concentrates (stem cell group). The first control group (control) included 40 patients underwent a vertical augmentation with autologous bone grafts from the iliac crest. In the second control group, 40 patients received identical surgical procedure, whereas the autologous bone graft was covered with a thin layer of deproteinized bovine bone matrix and a collagen membrane (DBBM group). Clinical complications, implant survival, radiological assessment of the stability of the vertical height and histological evaluation at the recipient site have been followed up for 24 months postoperatively.

**Results:**

No differences in terms of implant survival were observed in the groups. In the stem cell group, the resorption after 4–6 months was 1.2 ± 1.3 mm and significantly lower than the resorption of the control group with 1.9 ± 1.6 mm (*P* = 0.029) (DBBM group: 1.4 ± 1.2 mm). After 12 months, the resorption of the stem cell group was 2.1 ± 1.6 mm and significantly lower compared to the control group (4.2 ± 3.0 mm, *P* = 0.001) and DBBM group (resorption 2.7 ± 0.9 mm, *P* = 0.012). The resorption rate in the second year was lower compared to the first year and was measured as 2.7 ± 1.7 mm in the stem cell group (1-year bone loss in the time period of 12–24 months of 0.6 mm compared to 2.1 mm in the first 12 months). The resorption was significantly lower compared to the control group (4.7 ± 2.9 mm; *P* = 0.003, DBBM group: 3.1 ± 0.5 mm, *P* = 0.075).

**Conclusions:**

Autologous bone-marrow aspirate concentrate could enhance the dimensional stability of the bone grafts and improve the clinical standard of complex reconstruction of the alveolar ridge. Even though the intraoperative cell enrichment requires an additional equipment and technical specification, it represents an alternative method for in-situ regeneration by osteogenic induction with a contribution of a manageable cost factor.

## Background

Traumas, neoplasia requiring ablative surgery, infections, necrosis and severe atrophy due to tooth-loss could result in defects of the jaws that jeopardize implant-supported prosthetic rehabilitation due to the insufficient bone volume at the implant recipient site [1–4]. Despite recent developments in guided bone regeneration, distraction osteogenesis and tissue engineering applications, autologous bone augmentation presents still the gold standard in the reconstructive implant surgery [5–7].

The clinical outcome of autologous bone grafting depends on many factors, including type and fixation of the bone graft as well as both the donor and recipient sites. Depending on the volume of the osseous defect at the planned implant recipient site, the selected donor sites could vary. Whereas smaller, horizontal defects could be reconstructed with intraoral bone blocks and or cortical shields obtained from chin and ramal areas, two dimensional augmentations or large-volume defects could be reconstructed via iliac crest, calvaria or rib [8].

Thanks to its easy surgical access, low complication rates and sufficient bone augments containing both cortical and spongious structures, anterior iliac crest presents the best choice for the reconstruction of large alveolar bone defects [9]. However, the resorption of the bone graft, still presents a great challenge for the clinician, thus the dimensional instability could jeopardize the implant survival in long term [10].

In general, bone grafts have two main functions; they serve as a source of osteogenesis and as a mechanical support. An iliac bone graft—which contains both cancellous and cortical structures—provides a source of osteoprogenitor cells and is, therefore, osteo-inductive and therewithal acts as a load-bearing space filler thanks to its cortical components. However, it is well-known that, autologous bone grafts are initially resorbed; cancellous structures are completely replaced in time by creeping substitution, whereas cortical grafts remain an admixture of necrotic and viable bone for a prolonged period of time [11].

In the literature, it has been suggested that collagen membranes could reduce the post-operative resorption of the iliac bone graft, but the resorption rates are still 20% in the first year and up to > 30% after 5 years following augmentation [12]. Successful results were accomplished with deproteinized bovine bone matrix, with the hypothesis that bovine bone could be placed over grafted areas, taking advantage of its osteoconductive properties and compensating for the natural bone resorption caused by remodeling [13]. Marukawa et al. have showed that, autogenous cancellous bone grafting with platelet rich plasma could significantly reduce postoperative bone resorption [14]. Recently, Khoury and Hanser have described a tunnelling flap approach, which allows a hermetic soft tissue closure, an acceleration of transplant revascularization and long-term three-dimensional volumetric bone stability [15].

It is well-known that skeletal regeneration and repair are controlled by stem and progenitor cells [16]. In the literature, several studies have reported that, mesenchymal stem cells (MSCs) derived from bone marrow in combination of bone substitutes could support the regeneration of large bone defects and increase allograft osteointegration [17, 18]. Considering the mechanisms underlying the phenomenon “creeping substitution”, it would be of great interest if the dimensional stability of grafts from the iliac crest to reconstruct large alveolar defects could be ensued by autologous stem cells. Therefore, the objective of the current study was to assess the clinical and radiological outcomes following autologous grafting from the iliac crest treated with bone-marrow aspirate concentrate in-situ to reduce the postoperative bone graft resorption rate.

## Materials and methods

### Study design

The study group consisted of patients who underwent vertical augmentation of the jaws via bone grafts harvested from the iliac crest enriched with bone-marrow aspirate concentrate. The cohort was compared to two control groups, which were evaluated in a former study by the same institution. The first control group (Control) included 40 patients underwent a vertical augmentation with autologous bone grafts from the iliac crest, which were recruited by the Department of Oral and Maxillofacial and Plastic Surgery at Friedrich-Alexander-University Erlangen Nuremberg [19, 20]. In the second control group, 40 patients received identical surgical procedure, whereas the autologous bone graft was covered with a thin layer of deproteinized bovine bone matrix and a collagen membrane (DBBM-group) (Table 1) [20]. This cohort was recruited by the Department of Oral and Maxillofacial and Plastic Surgery, University Hospital Schleswig–Holstein, Campus Kiel. The consistence of the surgical procedures was secured as the main surgeon moved from Erlangen to Kiel.

**Table 1:** Description of the subgroups

	Patients (*n*)	Mean age	Range	Mandible	Maxilla	Mandible-Maxilla	Implants (*n*)
Stem cell	33	60	31–83	6	24	3	188
DBBM	40	64	22–80	8	26	6	248
Control	40	58	27–77	6	30	4	237
Total	113	60	27–83	20	80	13	673

Comparative assessment was conducted and reported in accordance with the STROBE recommendations (strengthening the reporting of observations in epidemiology). It was undertaken with the understanding and written consent of each subject. It was carried out according to the ethical principles including the Declaration of Helsinki. Ethical approval was obtained by the by local ethical committee (AZ D494/18).

The data of the patients who presented with critical size defects of the jaws who underwent vertical or vertical–horizontal augmentation via autologous bone graft harvested from anterior iliac crest were included. Patients with any systemic conditions which could affect the bone healing, such as: uncontrolled diabetes, smoking habits, immunosuppression, malignancies of the maxillofacial structures, antiresorptive therapy and radiotherapy to the head and neck region were excluded. Clinical and radiographic evaluation was carried out after 4–6 months postoperatively in 33 patients, 12 months postoperatively in 16 patients and 24 months postoperatively in 10 patients.

### Surgical procedure

**Fig. 1:**
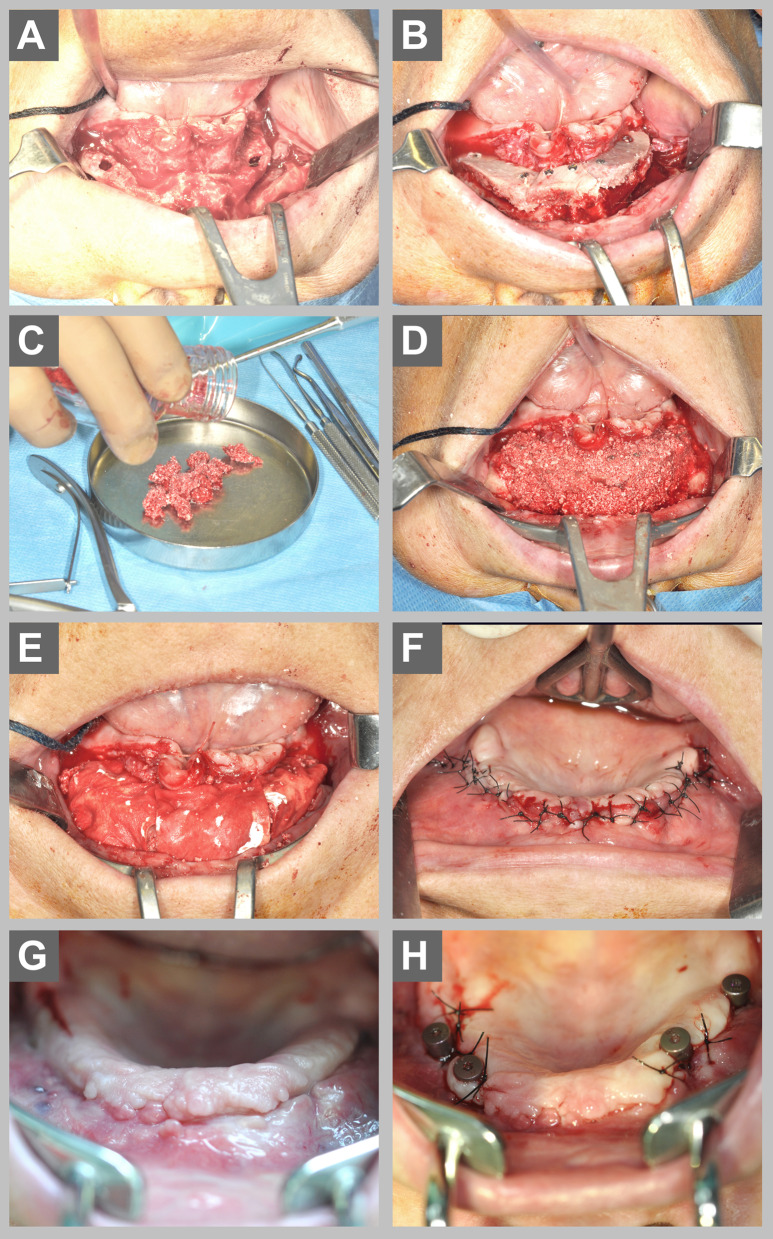
Intraoperative view on a severe atrophied maxilla (**A)** and the vertical augmentation with three blocks from the iliac crest, fixed with two screws each (**B**). The BMCA was mixed with autogenous bone chips and DBBM (**C)** to cover and contour the augmentation (**D**). The augmentation was covered with resorbable membranes followed by tension-free wound closure (**E**, **F**). Six months after the augmentation four implants were inserted (**G**, **H**)

**Fig. 2:**
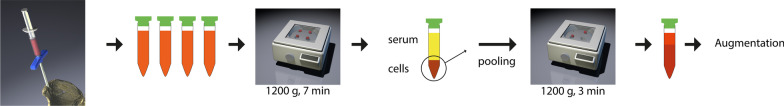
Schematic drawing of the preparation of enriched bone-marrow aspirate: bone marrow was aspirated with a bone cannula. 2500IE heparin per 20 ml syringe were added and a total of 50–60 ml of bone-marrow aspirate was gained. Centrifugation in 4 tubes at 1200 g for 7 min. Removal of the serum supernatant and pooling of the cell-concentrates in one tube for second centrifugation at 1200*g* for 3 min. Removal of the supernatant and approximately ten millilitres of enriched stem cells were gained

The augmentation procedure was performed as previously described by Wiltfang et al. [20]. Briefly, under general anesthesia, cortico-spongeous bone grafts were harvested from the anterior iliac crest. The grafts were trimmed according to the form and size of the implant recipient site and fixed with titanium mini screws (Fig. 1). If a sinus floor elevation was indicated, the previously described lateral window technique was used [21]. During the same procedure and via the same access, bone marrow was aspirated with a bone cannula (PrepaPlus®E 11G, Peter Pflugbeil GmbH, Zorneding, Germany), which was screwed into the iliac crest 8 cm posterior of the anterior iliac spina (Fig. 2). 2500IE heparin per 20 ml syringe were added and a total of 50–60 ml of bone-marrow aspirate was gained. The stem cell enrichment was performed chairside using a laminar airflow bench according to the following protocol: centrifugation in 4 tubes at 1200*g* for 7 min (Centrifuge 5702, Eppendorf SE, Hamburg, Germany), removal of the serum supernatant, pooling of the cell-concentrates in one tube for second centrifugation at 1200 g for 3 min. Finally, the supernatant was removed. Approximately ten milliliters of enriched stem cells were used for each recipient site. The stem cells were mixed with autogenous bone chips and DBBM to cover and contour the bone-blocks. The augmentation was covered with resorbable membranes (Bio-Gide®, Geistlich Pharma AG, Wolhusen, Switzerland). Ampicillin/sulbactam (3 × 1.5 g/day) was administered intravenously at the day of surgery and 2–3 days postoperatively and then continued as oral antibiotics (2 × 750 mg/day) for five additional days. The surgical wounds were sutured primarily closed layer-by-layer and in a tension-free manner. A postoperative “as-needed” analgesic regimen was performed. The implant insertions were made after 4–6 months.

### Clinical evaluation

**Fig. 3:**
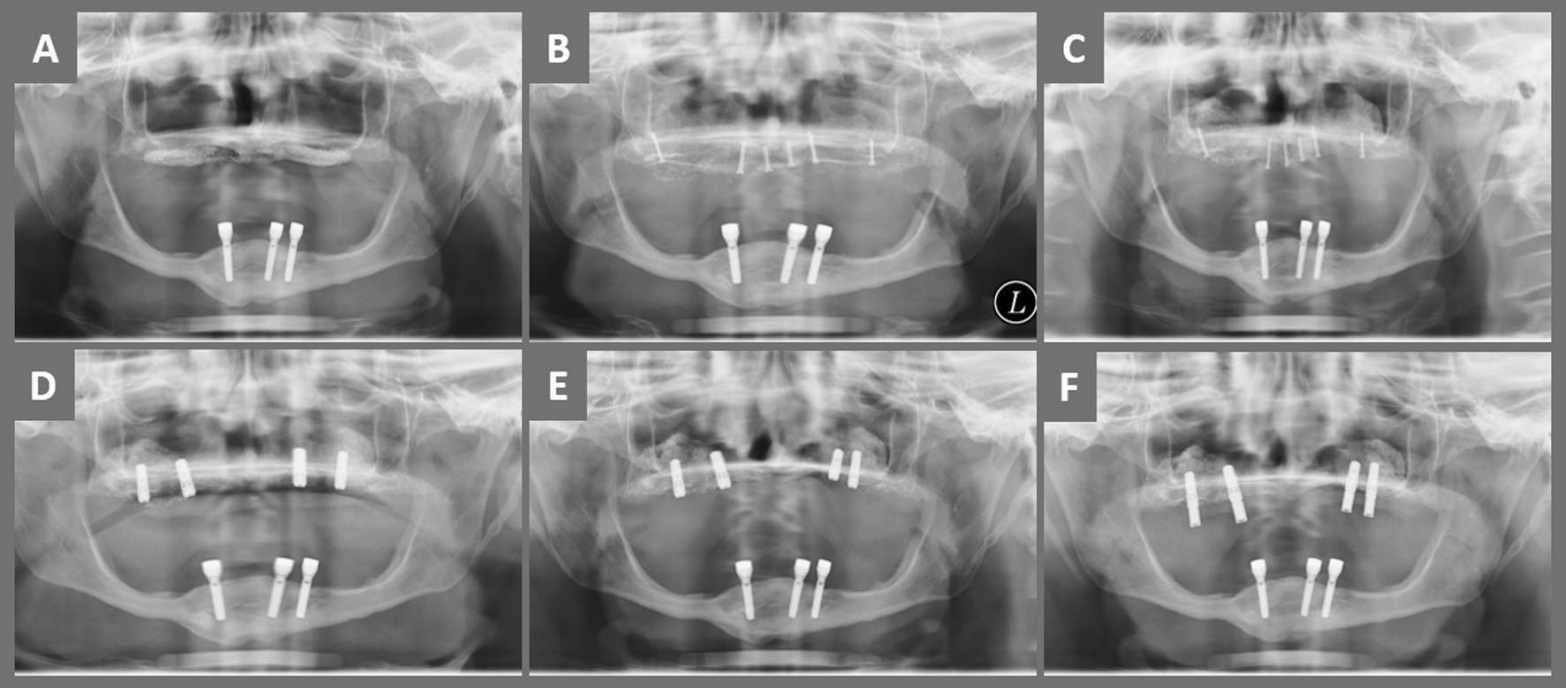
Panoramic radiographs: **A** preoperative, **B** immediately after augmentation, **C** after 3 months, **D** 5 months postoperatively after removal of the mini screws and implant insertion, **E** 11 months and **F** 23 months postoperatively

Clinical evaluation was carried out immediately after augmentation, after 6, 12 and 24 months postoperatively (Fig. 3). The evaluation included complications, such as wound dehiscence, abscess, transplant failure and implant survival.

### Radiographical evaluation

**Fig. 4:**
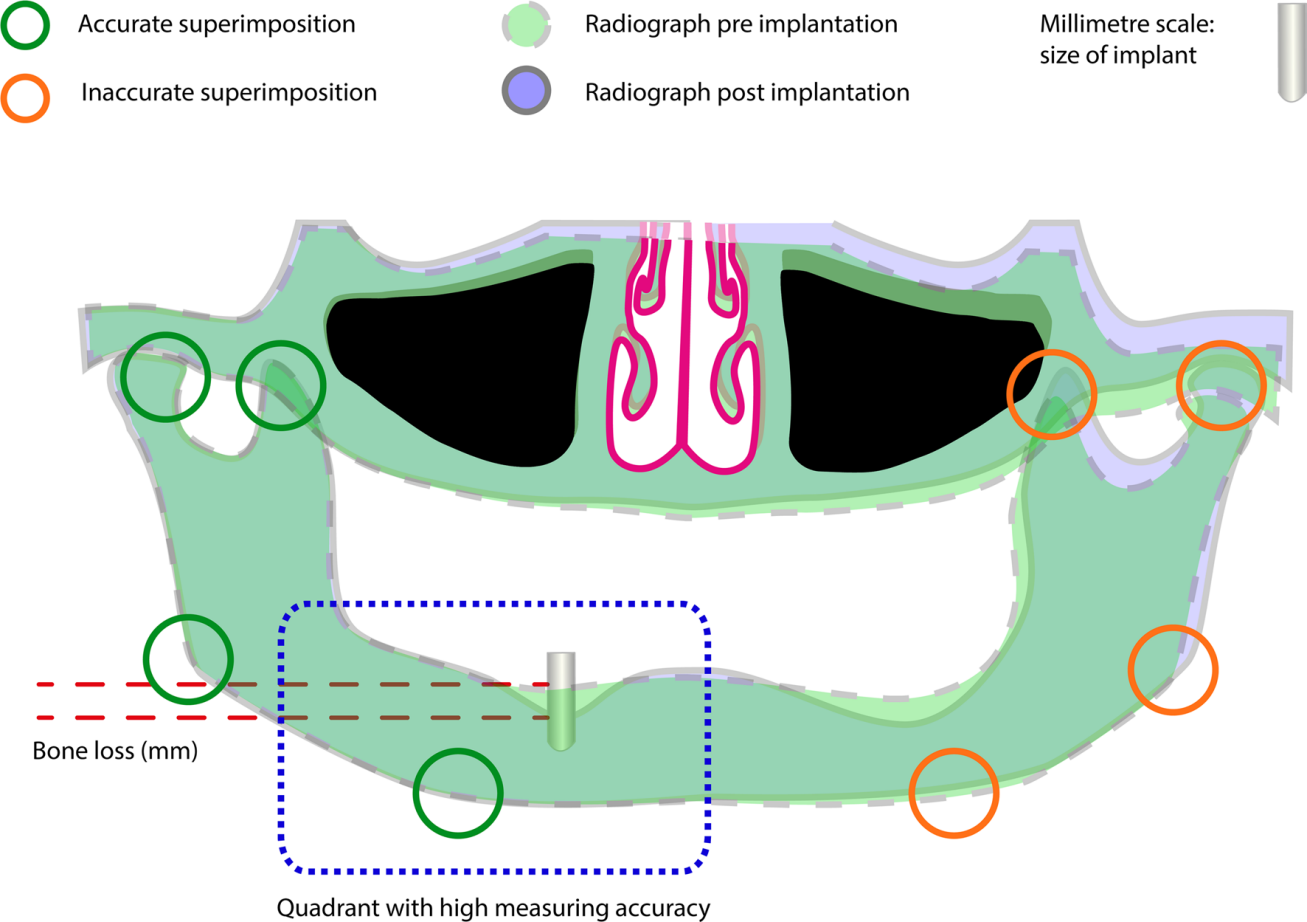
Superimposition and bone resorption measurement: specific marks in the radiographs (green circles) were used to achieve an accurate congruence of the pre and post implantation radiographs in the examined quadrant (blue box). The known size of an implant was used to set the millimetre scale and the time-dependent bone loss was measured (red-dashed lines)

Panoramic radiography was carried out with a ‘Kavo Pan eXam’ device (tube voltage: 66 kV (female patients) or 70–73 kV (male patients), current: 7.5–9.6 mA, exposure time: 10 s). The consecutive panoramic radiographs were processed by digital superimposition (Fig. 4). This method ensured the comparison of the bone height of each patients’ radiographs at different timepoints. By this method all radiographs of each patient were adjusted to each other for each single augmented quadrant. The different radiographs of one patient were opened in one file in several layers. The most recent served as a standard format. The other radiographs were adjusted to the standard format using a layer transparency of 40–45% and the formatting functions. This technique was performed for each single quadrant with augmented bone graft for a high degree of accuracy. The height of the bone was determined as the distance of the crestal edge of the residual alveolar bone and the graft surface. The known size of an implant in the examined quadrant served as a reference for the millimetre scale. The resorption was determined in millimetre compared to the initial height of the graft.

### Histological assessment

In ten patients treated with augmentation from iliac crest with additional BMCA, a specimen of the augmented bone was harvested using a 1.8 mm trepan bur at the time of implant insertion. The histological specimens of the control group included ten biopsies with conventional clinical standards with grafts from the iliac crest, absorbable membrane and particulate bone substitute material. The histological bone quality was evaluated using toluidine-blue staining. Clinical bone quality, density and structure, was assessed by intraoperative determination of the drilling resistance and classified by the Lekholm–Zarb classification system D1–D4 (D1: oak, D2: beech, D3: balsa, D4: polystyrene).

### Statistics

The resorption of the bone height was determined for each single augmented quadrant for each patient (63 quadrants after 4–6 months; 32 quadrants after 12 months; 25 quadrants after 24 months). The resorption was determined in millimetre and compared to the initial bone height. The mean resorption rate with standard deviation of all patients were calculated. The data was compared to the control groups. A two-sided *t* test was performed and *p* < 0.05 was considered significant.

## Results

The study group consisted of 33 patients (female: 24, male: 9) The mean age of the patients at the time of implantation of the bone graft was 60 years (range 31–81 years). 24 patients received bone graft augmentation in the maxilla, six patients in the mandible and three patients in both jaws.

### Clinical evaluation

**Fig. 5:**
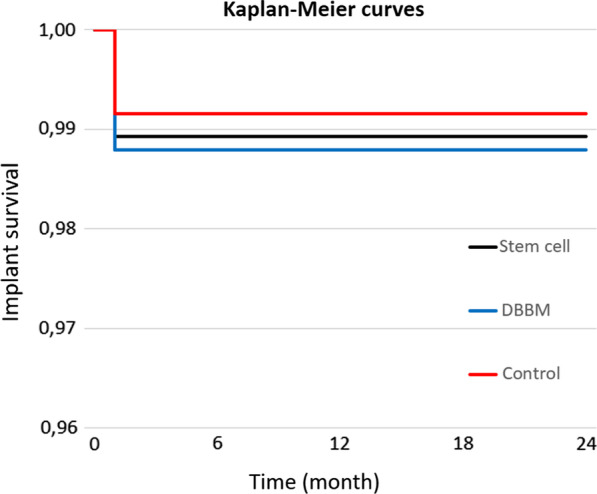
Kaplan–Meier curve for the implant survival of the three groups: seven of the 673 implants had to be removed due to failed osseointegration: two of 188 implants in the stem cell group, three of 248 implants in the DBBM group and two of 237 implants in the Control group

As the acquisition of the stem cells was performed ipsilateral to the donor site, patients did not report any additional discomfort caused by the aspiration–procedure. No differences in terms of implant survival were observed in the groups. No abscess or loss of bone graft occurred. In total, two out of 188 implants had to be removed due to failed osseointegration. With 186 implants successfully osseointegrated after 2 years the implant survival rate was 98.9% (DBBM: failed osseointegration of three of 248 implants, survival rate: 98.8%; control: failed osseointegration of 2 of 237 implants, survival rate 99.2%) (Fig*. **5*).

### Radiographical evaluation

**Fig. 6:**
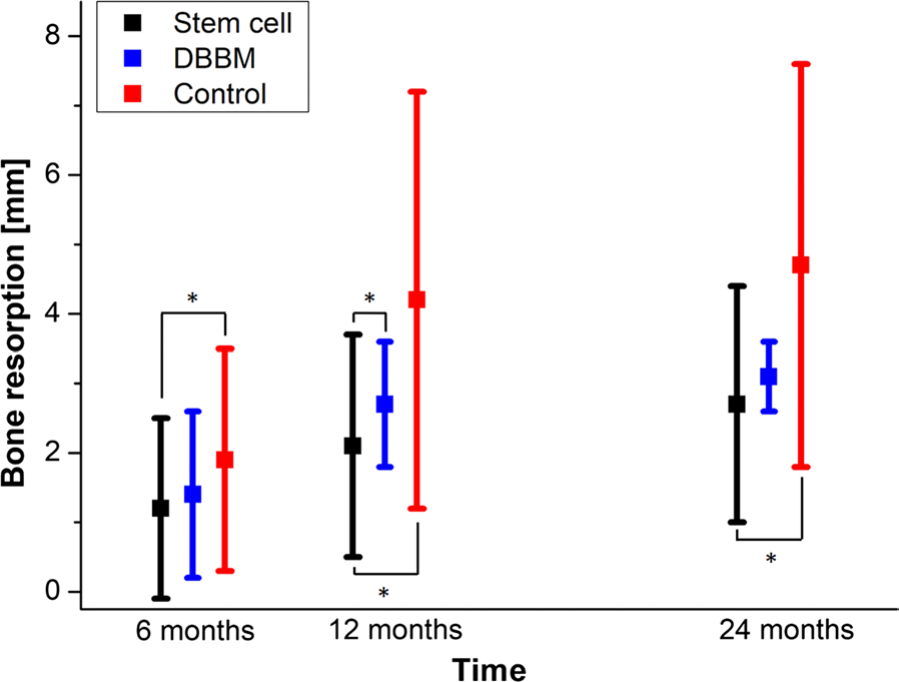
Bone resorption after 6, 12 and 24 months in the three groups: stem cell group (black); DBBM group (blue); control group (red). Significant differences are marked with an asterisk

In the study group, a total of 188 implants were inserted 4–6 months after augmentation. Patients of the control group received 237 implants, whereas patients of the DBBM group received 248 implants. Residual bone height at the augmented area was preoperatively 8.8 ± 2.7 mm. The average residual bone + graft height has reached to 14.2 ± 2.6 mm immediate postoperatively. The highest bone resorption rate was observed in the first 12 months postoperatively. In the stem cell group, the resorption after 4–6 months was 1.2 ± 1.3 mm and significantly lower than the resorption of the control group with 1.9 ± 1.6 mm (*P* = 0.029). The resorption in the DBBM group was a little bit higher but statistically not significant (1.4 ± 1.2 mm) (Fig. 6).

After 12 months, the resorption in the stem cell group was 2.1 ± 1.6 mm and significantly lower compared to the control group (4.2 ± 3.0 mm, *P* = 0.001) and significantly lower compared to the DBBM group (resorption 2.7 ± 0.9 mm, *P* = 0,012). The resorption rate in the second year was lower compared to the first year and was measured as 2.7 ± 1.7 mm in the stem cell group (1-year bone loss in the time period of 12–24 months of 0.6 mm compared to 2.1 mm in the first 12 months). The resorption was significantly lower compared to the control group (4.7 ± 2.9 mm; *p* = 0.003) but not significantly lower compared to the DBBM group (resorption 3.1 ± 0.5 mm, *P* = 0.075) (Fig. 6).

### Histological assessment

**Fig. 7:**
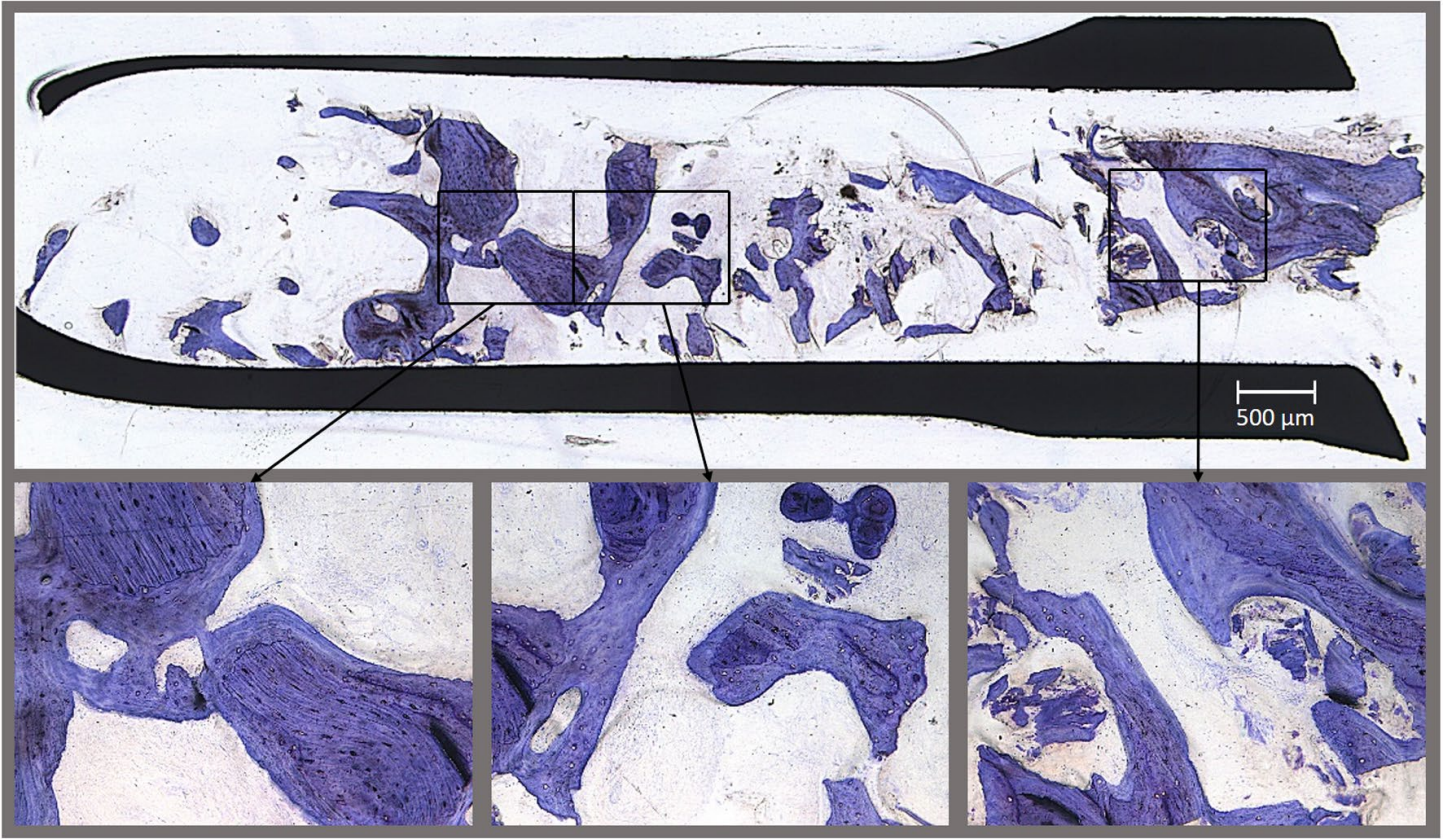
Toluidine blue staining of a bone biopsy of the upper jaw (still in the trephine bur) gained from a patient treated with enriched bone-marrow aspirate. The lower line shows magnifications of the regenerated bone, the areas are indicated by the black boxes in the overview

The clinical and histological bone quality were evaluated at the moment of implant insertion 4–6 month post-augmentation. Relative amounts of bone area vs total tissue of the spongious bone were measured (Fig. 7) [22]. The measured mean bone area (bone density) was 27.3 ± 10.9% in the stem cell group compared to 20.3 ± 13.4% in the control group. A higher clinical bone density (D1, 8, vs D2, 4) of the transplants with enriched bone-marrow aspirate treatment (*p* = 0.02) correlated with a better histological bone quality (*p* = 0.03).

## Discussion

The speed of graft remodeling—which depends on the type of the graft and its properties—may influence the final outcome of a restoration, thus an implant-supported prosthetic restoration may produce the worse-case-scenario if the graft remodeling was not completed [23, 24]. Therefore, the resorption of bone grafts is a clinical problem which may compromise the final outcome of dental implant therapy both from the aesthetic and functional standpoints. Therefore, the current emphasis is placed on the potential benefits of autologous mesenchymal stem cells (AMSCs) obtained via bone-marrow aspirate concentration in ensuring the dimensional stability of bone blocks at the edentulous ridges and clinical outcomes of dental implant therapy.

It has been observed that bone blocks treated with enriched bone-marrow aspirate showed a significant superiority in terms of dimensional stability both after 12 and 24 months compared to DBBM and control groups. However, an important point, which should not to be overlooked is that the resorption was observed most prominently after 12 months. A literature survey has confirmed different remodeling rhythms depending on the graft material and showed that greater dynamics of bone remodeling after augmentation with autologous bone grafts [25]. Deluiz et al. evaluate the behavior of bone block allografts for the alveolar ridge augmentation in two different healing timepoints and indicated that there is a significant difference regarding the resorption of the grafts when waiting 4 or 6 months before placing the implants, even though no difference was found in the histological, histomorphometric, and immunohistochemical features [24]. Considering the peak point of the resorption 12 months postoperatively and the above-mentioned fact that the implant insertion would be more suitable after 4 months, the dilemma of the ideal time for implant insertion regarding the remodeling phase, still exists.

A recent experimental study has demonstrated that combinations of β-TCP and DMP1 gene-modified MSCs could be used to construct tissue-engineered bone to enhance mineralization of the regenerated bone and osseointegration of dental implants [26]. Promotion of new bone formation and maturation via MSCs has been also shown previously [27]. Current study has also particularly showed that AMSCs treatment could increase the histological density of the augmented area; however, we think that the difference would not influence the treatment modality. However, a further study might focus on the effect of this difference in terms of mechanical anchorage of the dental implant and thereby assessing the possibility of an immediate loading protocol [28, 29].

It is well-known that the interaction of the host and the bone graft determines the success of the bone grafting procedure, which ultimately is to provide a mechanically efficient support structure [11]. Therefore, cortical bone grafts, which could provide both the most desirable osteogenic properties and a long-term dimensional stability, are the first choice in onlay-grafting of the atrophied edentulous alveolar ridge. However, the phenomenon “creeping substitution” which could be determined as the gradual replacement of the bone grafts by the cartilage and afterward with bone, which involves approximately 30% of the volume of graft is unfortunately not completely avoidable [30]. Several techniques have been proposed to overcome this process. It is well-known that vascularized bone grafts are capable of primary bony healing without creeping substitution and can replace deficient bone; however, the donor site morbidity and complication rates present still a great challenge for the clinician. Wiltfang et al. showed that the coverage of the iliac bone block grafts via deproteinized bovine bone matrix could reduce the affected volume of the graft during this process and allows more dimensional stability at the augmented area [20]. On the other hand, Giudice et al. have proclaimed that the use of platelet products in alveolar bone grafting could accelerate “creeping substitution” process and favors earlier second-stage treatment [31]. Considering the need of completion of the dental implant therapy to enhance a volumetric stability during the ongoing unavoidable remodeling process, the question to be answered should be:

- To reduce, or to accelerate the creeping substitution?

The idea of the use of AMSCs obtained via bone-marrow aspirates and concentrates in alveolar bone grafting is not a novel idea. In the literature, several articles have focused on the use of AMSCs in different pre-implantological treatment routines for alveolar ridge atrophy with various results [32]. Kühl et al. added bone-marrow aspirates and concentrates to deproteinized bovine bone mineral and investigated the grafts stability during the first 6 months after maxillary sinus lift augmentation [33]. They stated that bone-marrow aspirate or concentrate does not influence the dimensional stability of the bone grafts. Wildburger et al. have investigated on a split mouth design seven patients with a bilateral highly atrophic posterior maxilla and placed xenografts with and without AMSCs [34]. During a follow-up period of 3–6 months, no significant differences in bone formation between the both groups could be observed. Rickert et al. showed significantly more bone formation in bovine bone mineral seeded with AMSCs from the iliac crest compared to bovine bone mineral mixed with autologous bone [35].

From the financial perspective, the chairside method to harvest AMSCs is suggested to be a viable option for enhancing bone volume at the implant recipient site. The use of the technique might create a false perception of an increase in financial costs due to the need for special equipment and kits, thus the chairside method to harvest bone-marrow aspirate concentrates were often confused with bone-marrow-derived mononuclear cell isolation by synthetic polysaccharide (FICOLL). FICOLL is stated to be an optimal approach for harvesting of mononuclear cells [36]; however, it requires good manufacturing practice laboratory techniques with additional cost and time. In addition, no differences could be detected regarding the implant survival rates between chairside AMSC harvesting and FICOLL [37].

Considering the assessment of the bone grafting procedures, Abidi et al. suggested that, incremental costs associated with iliac crest autograft begins at. $1,465 USD and can even be higher [38]. Allograft cancellous chips combined with a bone-marrow aspirates costs significantly less with average pricing for allograft cancellous chips at $242 for 15 cc (2013 Spinal Surgery Update. Orthopedic News Network (ONN), 24(4), October 2013.) and a bone-marrow aspirate kit for chairside harvesting of AMCSs at $175. (Internal data, Biomet Biologics, 2014/The Use of Bone-Marrow Aspirate in Bone Grafting: A Value Proposition (zimmerbiomet.com)) According to a review of the costs for bone substitutes, average selling price for 10 cc of growth factor product is $5,000, DBM putty $1,531, bone graft substitutes are $1,994, and allogeneic cell-based matrices is $4,223. (US Markets for Orthopedic Biomaterials 2014, RPUS20OB13, Millennium Research Group, November 2013.) According to the above-mentioned financial aspects, harvesting of ASMCS require special equipment, but additional costs are substantially lesser than the use of bone substitutes and it contributes a manageable cost in autologous bone grafting.

Distraction osteogenesis—which has been mainly proposed to overcome a donor site morbidity—could be a feasible alternative to AMSCs treated bone grafting. Moreover, it has been proclaimed that vertical osseous enhancement obtained by distraction osteogenesis could provide more stability. However, a recent article has shown that both distraction osteogenesis and autogenous block grafting for vertical bone augmentation presented with similar results in terms of dimensional stability [39].

Difficulties in the evaluation of the radiographs arise due to the projection of a three-dimensional bone structure onto a two-dimensional image. A perfect congruence of the radiographs at different times could only be achieved with the exact same position of the head during the orthopantomography for all scans. Minor inclination of the head and, therefore, the projection angle, the distance between patient and orthopantograph, as well as the height of the device, influences the result of the panoramic radiograph. However, superimposition is an established method to accomplish congruency for certain region of interest, and hence, determine the time-dependent bone loss for each quadrant. The assessment of the dimensional stability only in vertical direction could be the main limitation of the current study. To avoid a radiation exposure secondary to cone–beam CTs during the whole follow-up period, panoramic radiographs have been used as a radiological tool not only in this but also in previous studies as a clinical standard.

## Conclusions

Additional application of bone-marrow aspirate concentrate might improve the clinical standard of complex reconstruction of the alveolar ridge and help to reduce the postoperative resorption rate and increase density and stability of the regenerated bone by osteogenic induction.

## Data Availability

The data sets used and analysed during the current study are available from the corresponding author on reasonable request.

## Abbreviations

DBBM: Deproteinized bovine bone matrix
STROBE: Strengthening the reporting of observations in epidemiology
AMSCs: Autologous mesenchymal stem cells
β-TCP: β-Tricalcium phosphate
DMP1: Dentin matrix acidic phosphoprotein 1
MSCs: Mesenchymal stem cells
CT: Computed tomography
